# Peculiarities of Thermodynamic Behaviors of Xenon Adsorption on the Activated Carbon Prepared from Silicon Carbide

**DOI:** 10.3390/nano11040971

**Published:** 2021-04-09

**Authors:** Ilya Men’shchikov, Andrey Shkolin, Elena Khozina, Anatoly Fomkin

**Affiliations:** M.M. Dubinin Laboratory of Sorption Processes, A.N. Frumkin Institute of Physical Chemistry and Electrochemistry (IPCE RAS), Russian Academy of Sciences, Leninskii prospect, 31, Str. 4, Moscow 119071, Russia; shkolin@bc.ru (A.S.); elena-khozina@rambler.ru (E.K.); fomkinaa@mail.ru (A.F.)

**Keywords:** xenon, activated carbon, adsorption, adsorption-induced deformation, thermal expansion, thermodynamics of adsorption

## Abstract

An activated carbon prepared from silicon carbide by thermochemical synthesis and designated as SiC-AC was studied as an adsorbent for xenon. The examination of textural properties of the SiC-AC adsorbent by nitrogen vapor adsorption measurements at 77 K, powder X-ray diffraction, and scanning electron microscopy revealed a relatively homogeneous microporous structure, a low content of heteroatoms, and an absence of evident transport macropores. The study of xenon adsorption and adsorption-induced deformation of the Si-AC adsorbent over the temperature range of 178 to 393 K and pressures up to 6 MPa disclosed the contraction of the material up to −0.01%, followed by its expansion up to 0.49%. The data on temperature-induced deformation of Si-AC measured within the 260 to 575 K range was approximated by a linear function with a thermal expansion factor of (3 ± 0.15) × 10^−6^ K^−1^. These findings of the SiC-AC non-inertness taken together with the non-ideality of an equilibrium xenon gaseous phase allowed us to make accurate calculations of the differential isosteric heats of adsorption, entropy, enthalpy, and heat capacity of the Xe/SiC-AC adsorption system from the experimental adsorption data over the temperature range from 178 to 393 K and pressures up to 6 MPa. The variations in the thermodynamic state functions of the Xe/SiC-AC adsorption system with temperature and amount of adsorbed Xe were attributed to the transitions in the state of the adsorbate in the micropores of SiC-AC from the bound state near the high-energy adsorption sites to the molecular associates.

## 1. Introduction

Xenon is the least abundant of the noble gas family of elements. It is found in trace amounts in the Earth’s atmosphere (8.6 × 10^−5^ vol. %) and also as a component of gases evolved from mineral springs [1,2]. Xenon is obtained in industrial-scale volumes as an air separation by-product at the large-scale production of liquid oxygen at metallurgical plants [3]. In a typical industrial setup, the gas is separated from a krypton–xenon pre-concentrate by low temperature (cryogenic) rectification, providing xenon of high purity (99.9999%). It should be noted that before this stage, adsorption technologies are employed for deep purification of the mixture. 

On a much smaller scale, xenon and krypton are emitted as fusion products of uranium dioxide used as fuel for reactors at the existing nuclear power plants (NPP). The emissions of operating Russian NPPs consist of 99.9% inert radioactive gases [4]. The fission gases are dominated by Xe and Kr, which exist in the form of approximately 20 isotopes. ^88^Kr, ^133^Xe, and ^135^Xe are the dominant isotopes with half-lives of 2.8 h, 5.3 days, and 9.2 h, respectively. The relative amounts of the xenon isotopes are somewhat dependent upon the kind of reactor. In some types of nuclear reactors, it is necessary to remove ^135^Xe from the cooling gas for reasons of neutron economy since ^135^Xe has a very large neutron capture cross-section [5]. On the other hand, separation and capture of xenon from krypton in the off-gas flow would provide a new resource of xenon for industrial use.

Indeed, high purity xenon has applications in a wide range of fields, including medicine, industry, and science. It is used in high-pressure arc lamps for motion picture projection and producing ultraviolet light. Xenon (^127^Xe, ^133^Xe, and ^137^Xe) is utilized in radiation detection devices, e.g., X-ray and neutron counters. The potential applications of liquid xenon as a detection medium in medical diagnostic imaging systems with radioisotopes have been explored since the 1970s [6]. Xenon is a highly effective neuro-protector; it is considered to have ideal anesthetic properties due to a fast response and no relevant side effects [2]. Inert gases—in primis xenon—are used in modern ion thrusters for space travel, namely, for propellants to avoid the risk of explosions, which are incidental to chemical propulsion [7]. 

In Russia and the Commonwealth of Independent States (CIS) countries, approximately 1500 m^3^ of the standard temperature and pressure conditions (STP) of xenon is produced annually [8]. The annual industrial production of xenon in the world is close to 10,500 m^3^ (STP) [9]. According to analytical forecasts [10], the global xenon market exhibits a compound annual growth rate of 1.7% during the years 2020–2027. The driving factors of the xenon market are as follows:

- Increasing utilization of electric propulsion systems in satellite and rocket launches;
- Growing demand for anesthetic in the medical industry;
- Widening applicability in electronics and semiconducting industry;
- Massive employment in the R&D projects, including dark matter investigations.

Cryogenic rectification noted above is currently the most ubiquitous technology to separate Kr and Xe from the air. Since this method involves multiple heating, cooling, and pressurization processes, it is energy-intense and therefore expensive. Indeed, the cost of Xe is extremely high (USD 10.00–18.00 per liter at standard temperature and pressure [11]). Moreover, the significant energy consumption and the resultant emission of carbon dioxide upon production certainly diminish the environmental advantage of xenon [12]. These factors actuate the development of an alternative technology for a less energy-intense, more economically viable, and safer process to produce xenon, not de novo but by retrieving from waste gas (i.e., recycling). 

Although xenon has the chemical properties of non-polar and uncharged noble gases, it can bind via van der Waals forces as an instantaneously polarized atom. The high number of electrons and low binding energy are responsible for the ability of xenon to be polarized, which is the greatest among other noble gases. Therefore, a promising alternative technology for Xe concentrating and storage in a recycling system, including its capture and selection from the nuclear reactor reprocessing off-gas or waste anesthetic gas, is a physical adsorption-based process, which can be carried out at room temperature using an effective adsorbent [5,13]. The efficiency of xenon adsorption technologies depends significantly on the porous structure and surface chemistry of an adsorbent, and the thermodynamic behaviors of an adsorption system (changes in the heat of adsorption, enthalpy, entropy, and heat capacity) under prescribed operating conditions (temperature and pressure). 

The theory of volume filling of micropores (TVFM) developed by Dubinin [14,15] asserts that adsorption capacity for any substance depends on the structural and energy parameters of a microporous solid: micropore volume and characteristic energy of adsorption. Experimental studies of xenon adsorption on activated carbons [5,16,17,18,19], zeolites [20,21], silica gels [5,22], metal-organic framework structures (MOFs) [19,22,23,24,25,26,27], and porous organic cages [19,23,24,25,26] revealed that these adsorbents showed high capacity and sufficient selectivity toward Xe over Kr. Although MOFs show potential as an adsorbent for the Xe adsorption-based technologies [22,23,24,25,26,27], there are some constraints in their large-scale production and applications, among which are relatively low reproducibility between different batches of final product and high production costs [28,29]. Moreover, highly polarizable water molecules interact with open metal sites in MOF strongly than xenon atoms do and can therefore be first adsorbed on the open metal sites, diminishing the total uptake [23,24]. 

At the same time, it should be noted that in addition to a relatively high adsorption capacity in respect to xenon (up to 50 wt. % at 1 bar and 298 K [17,24]), activated carbons (ACs) are the commercially viable materials for various technologies based on or attended by adsorption processes due to their chemical and thermal stability, mechanical strength, the abundance of carbon-rich raw materials, and the availability of mature methods of their large-scale production [30,31]. Among the properties of ACs, which are essential for the gas adsorption-based technologies, mention should be made of their high adsorption capacity per unit volume, low resistance to gas flow, hydrophobicity, and exhaustive release of adsorbates with increasing temperature and decreasing pressure [30]. 

In our study, we examined the factors affecting the xenon adsorption performance of a carbon adsorbent prepared from silicon carbide, SiC-AC. To do so, we first considered the data on its structural and energy characteristics, chemical composition, and morphology obtained by the standard method of nitrogen adsorption at 77 K, X-ray diffraction (XRD), and scanning electron microscopy (SEM). Second, we focused on measurements of xenon adsorption in Si-AC over a wide range of temperatures and pressures, which provided us a possibility to calculate the thermodynamic characteristics of the adsorption system. Our analysis rests on an approach elaborated by Bakaev [32], which originated from Guggenheim’s concept of rigorous thermodynamics of adsorption equilibrium, which implies that the thermodynamic functions of an adsorption system are evaluated from the experimentally measured quantities [33,34,35]. This approach circumvents the difficulty in interpreting adsorption data coming from the Gibbs formalism, which considers adsorption phenomena in terms of interfacial excess amounts [32]. Moreover, this framework offers a practical way of calculating the thermodynamic functions of an adsorption system from the experimental isosteres of adsorption with consideration of the impacts from the non-ideality of an equilibrium gas phase and non-inertness of an adsorbent at high pressures and temperatures [32]. With these considerations in mind, we measured the changes in the sizes of the SiC-AC sample induced by xenon adsorption and also by temperature with the purpose to evaluate properly the thermodynamic state functions of the xenon/SiC-AC adsorption system, namely the heats of adsorption, enthalpy, entropy, and heat capacity. In conclusion, we examined the influence of the amount of adsorbed Xe and temperature on the thermodynamic properties of the Xe/SiC-AC adsorption system.

## 2. Materials and Methods

### 2.1. Adsorbent

The SiC-AC carbon porous material was prepared from α-silicon carbide by leaching of Si atoms in a chlorine atmosphere at 1173 K. The thermochemical reaction produced volatile SiCl_4_ gas and a rigid monoporous carbon matrix [36]:

SiC + 2Cl_2_ → SiCl_4_ + C, (1)

**Figure 1:**
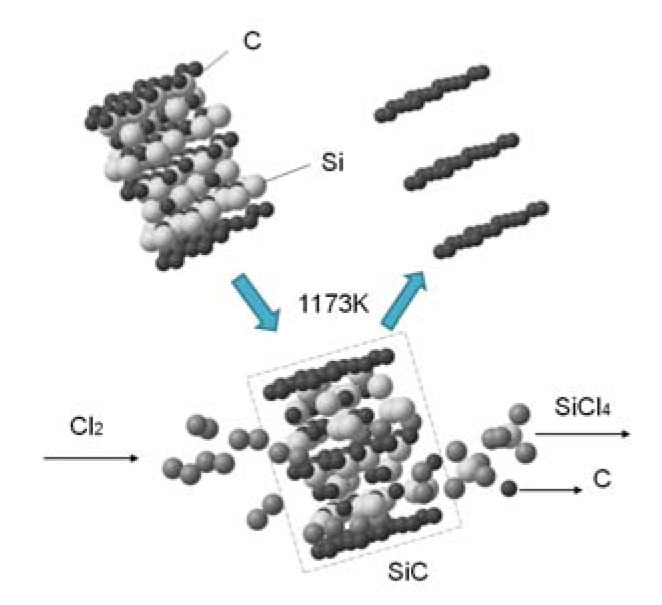
Scheme of the formation of a fragment of the silicon carbide (SiC-AC) carbon adsorbent via thermochemical leaching of Si atoms from silicon carbide.

Figure 1 illustrates the formation of a porous carbon matrix of SiC-AC schematically as a result of the thermochemical reaction.

### 2.2. Adsorptive

Ultra-high purity grade 6.0 (99.9999%) xenon was used for adsorption measurements. The physicochemical characteristics of xenon are as follows: molecular mass µ = 131.293 g/mol; critical pressure *p*_cr_ = 5.842 MPa; critical temperature *T*_cr_ = 289.73 K; and normal boiling point *T*_0_ = 165.05 K [37]. 

### 2.3. Methods

The porous structure parameters of the SiC-AC adsorbent were deduced from the standard nitrogen adsorption isotherm measured at 77 K by a Quantachrome Autosorb iQ multifunctional surface area analyzer. 

The constitutional equation of TVFM, namely, the Dubinin–Radushkevich (D–R) equation [14,15] was employed to calculate the structural and energy characteristics, viz., micropore volume (*W*_0_), characteristic energy of adsorption (*E*_0_), and effective half-width of micropores (*x*_0_) of the SiC-AC sample:

(2) $$a=a_{0}\exp\left[-{\left(A/E\right)}^{2}\right],$$

where $A=RTln\left(P_{s}/P\right)$ [kJ/mol] is the differential molar work of adsorption; *P*_s_ is the saturation pressure; and *a*_0_ [mmol/g] is the limiting value of adsorption at the temperature *T* [K], which is a function of *W*_0_. For a model of slit-like pores, which are typical for activated carbons, the characteristic energy of adsorption is related to the effective half-width of micropores by a formula: *E* = 12β/*x*_0_, where β is the coefficient of similarity for the gas under study. The value of β is evaluated relative to the standard benzene vapor: β = *E*/*E*_0_.

The specific surface area (*S*_BET_) of the SiC-AC adsorbent was estimated using the classic Brunauer–Emmet–Teller (BET) [38] equation. When determining the pore size distribution in SiC-AC from the nitrogen adsorption isotherm, we addressed the non-local density functional theory (NLDFT) applied for a combined slit + cylindrical pore model [39].

Information on surface morphology and chemical composition of the mechanically disintegrated SiC-AC material was obtained by using a Quanta 650 FEG scanning electron microscope (FEI, Company, Hillsboro, OR, USA) equipped with an Oxford Inca energy-dispersive X-ray (EDX) system. The elemental composition of the SiC-AC sample was evaluated as an average of at least ten scans.

The phase composition of the SiC-AC sample was inferred by the powder XRD pattern recorded with an Empyrean (Panalytical BV) diffractometer in Bragg–Brentano geometry using a nickel-filtered CuKα (λ_Cu_ = 0.1542 nm) radiation in the 2θ angular range of 10° to 120°. The sample was powdered, and we did not use any binder. Phase identification was carried out employing the International Centre for Diffraction Data PDF-2 (ICDD PDF2) database. The characteristic graphite reflections (002), (10), (100), (101), and (11) were used for qualitative analysis. 

Xenon adsorption equilibria on the SiC-AC adsorbent were studied in the temperature range of 178 to 393 K and at a pressure as high as 6 MPa using two adsorption instruments engineered in IPCE RAS [40,41]. A semi-automatic gravimetric vacuum setup [40] enabled us to measure xenon adsorption at pressures up to 0.1 MPa (1 bar) with the combined standard and expanded uncertainties of ±1.4 and ±4.0%, respectively [42]. Xenon adsorption measurements were carried out within the range of 0.1 (1 bar) to 6.0 MPa by a universal volumetric adsorption-dilatometer setup [41]; the combined standard and expanded uncertainties amounted to ±3.1 and ±12.0%, respectively. 

Before the experiments, the SiC-AC sample was evacuated at 573–623 K for 6 h up to the pressure of 0.1 Pa. In the subsequent discussion, we analyzed the value of absolute xenon adsorption on SiC-AC, which was calculated as an amount of gas adsorbed from a measuring unit, adjusted for a skeletal volume of the adsorbent determined from the helium pycnometry experiments [43], *V*_He_ [cm^3^], and micropore volume, *W*_0_ [cm^3^/g], evaluated from the nitrogen adsorption isotherm at 77 K by the D–R equation (2). Thus, the absolute xenon adsorption on the SiC-AC adsorbent was calculated as follows:

*a* = (*N* − (*V* − *V*_a_) × ρ_g_)/(μ×*m*_0_). (3)

where *N* is the amount of xenon introduced into a measuring unit, [g]; *V* is the total geometric volume of the measuring system, [cm^3^]; *V*_a_ is the volume of an adsorbent with micropores, [cm^3^] calculated as a sum, *V*_He_ + *m*_0_ × *W*_0_, [cm^3^]; ρ_g_ is the density of a gaseous phase, [g/cm^3^] at specified pressure *P* and temperature *T*; μ is the molar mass of gas, [g/mmol]; and *m*_0_ is the mass of a regenerated adsorbent, [g]. The relative volumes of meso- and macropores, and, consequently, their contributions to the total adsorption process, as reported below, were negligible. 

The adsorption- and temperature-induced deformation of the SiC-AC adsorbent was measured using an induction dilatometer that allowed measurements of adsorbent deformations up to the pressure of 20 MPa and within the temperature interval of 77 to 670 K. The range of measured values of absolute deformation was 10^−7^–10^−3^ m. The setup scheme is given [41], and a procedure of measurements was described in detail in our previous studies [41,44]. Here, we highlight only some key items, which are essential for the validation of the measurements:

1. A system calibration algorithm, which takes into account a deformation effect of the setup itself induced by xenon adsorption or temperature under experimental conditions, was employed. The dilatometer was calibrated using a fused quartz mockup, the shape and size of which were identical to that of the tested SiC-AC sample—a rod, which was 54.0 mm long and 11.4 mm in diameter. Quartz was selected due to its low compressibility. Calibration curves were recorded with a gradual increase in pressure up to 6 MPa (or temperature from 213 to 573K) and used for evaluating the systematic amendments;
2. An adsorption stabilization procedure of the SiC-AC sample consisting of a gradual increase in xenon (or nitrogen for thermal deformation measurements) pressure up to 6 MPa, holding for 30 min, followed by xenon export using a vacuum pump. A sequence of ten adsorption/desorption cycles made it possible to verify the reversibility of adsorption (or temperature)-induced deformations of SiC-AC. Before each measurement, the adsorbent was regenerated at 623 K for 2 h to the pressure of 0.01 Pa;
3. The signal stability was ensured by storing the measuring units for 3 h at each experimental temperature. The dilatometer unit containing the SiC-AC sample was thermostatted at each experimental temperature with an accuracy of 0.2 K.
4. Adsorption-induced deformation was measured in the temperature range of 216 to 393 K and at pressures varying from 0.1 to 6 MPa. Thermal deformation of SiC-AC was measured within the range of temperatures of 260 to 575 K.

Thus, a fractional change in the linear dimension of the SiC-AC adsorbent driven by xenon adsorption, η*_a_*, was calculated as follows:

(4) $$\eta_{a}=\frac{\Delta l}{l_{0}}=\frac{\left(\left(U_{1}-U_{0}\right)-\left(U_{3}-U_{2}\right)\right)K_{L}}{l_{0}},$$

where ∆*l* is the absolute variation in the length of the SiC-AC sample; *l*_0_ is the initial length of the SiC-AC sample; *U*_1_ and *U*_0_ are the current and initial readings of a differential transformer of the dilatometer with the SiC-AC adsorbent, respectively; *U*_3_ and *U*_2_ are the current and initial readings of the differential transformer of the dilatometer with the quartz mockup, respectively; and *K_L_* is the conversion factor for evaluating the linear displacement from the change in voltage. 

The linear thermal expansion factor of the adsorbent, α, was evaluated from a formula:

(5) $$\alpha =\frac{d\eta_{T}}{dT}=\frac{1}{\Delta T}\frac{\Delta (\left[\left(U_{1}-U_{0}\right)-\left(U_{3}-U_{2}\right)\right]K_{L})}{l_{0}}$$

where η*_T_* is a relative change in the linear dimensions of the SiC-AC sample caused by temperature. Evidently, the largest error in the estimated adsorption-induced deformation may occur at small changes in the adsorbent size when the difference between *U*_1_ and *U*_0_ is relatively small. The calculations revealed that the distribution of errors adheres to the near-normal law. Thermal- and adsorption-induced deformations were measured within a 95% confidence interval with the confidence limits ∆η = ± 5.23·10^−7^ m, determined according to ISO 11095:1996 statistical methods. Linear calibration using reference materials [45]; the standard deviation was 3.7%.

## 3. Results

### 3.1. Structure and Morphology Characterization of the SiC-AC Adsorbent

**Figure 2:**
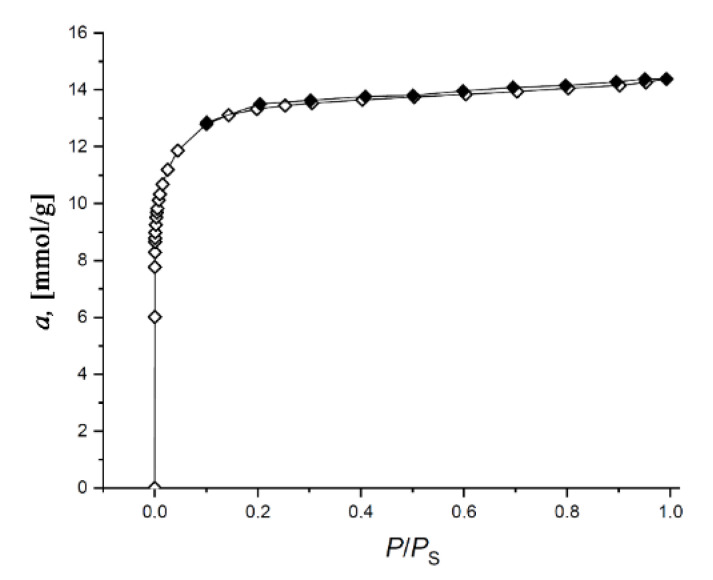
Isotherm of nitrogen adsorption (empty symbols)/desorption (dark symbols) on the SiC-AC carbon adsorbent measured at 77 K. Solid lines are the results of approximation by the Dubinin–Radushkevich (D-R) equation.

Figure 2 shows the Γ-shaped (or Type I) isotherms of nitrogen adsorption/desorption at 77 K on the SiC-AC adsorbent, which are typical for microporous solids [46]. 

**Figure 3:**
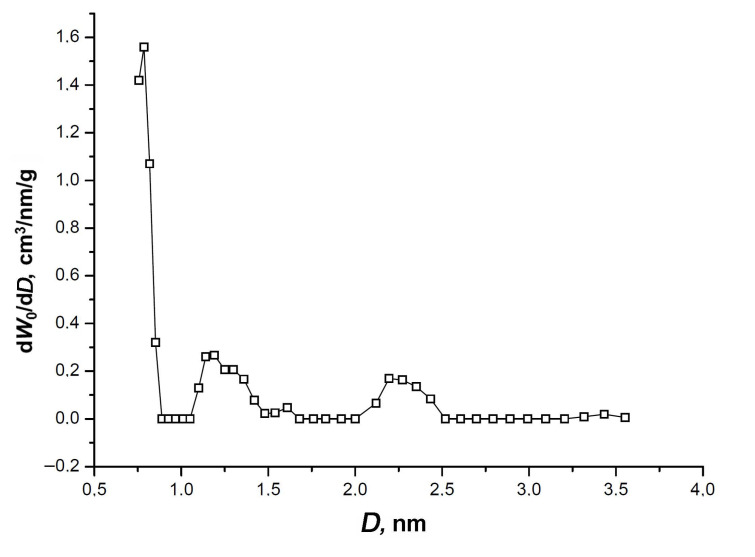
The pore size distribution of SiC-AC calculated by the non-local density functional theory (NLDFT) model for a combined slit + cylinder pore geometry from the nitrogen adsorption isotherm at 77 K [39].

The isotherm exhibits a narrow hysteresis loop, which is indicative of an insignificant fraction of transport mesopores displayed by a low-intensity broad peak at 2.2–2.3 nm on the pore size distribution shown in Figure 3. 

Figure 3 also shows one sharp pronounced maximum of the micropore size at 0.7–0.8 nm, and a low-intensity peak at *D* = 1.2 nm. It should be noted that the NLDFT model data match the parameters of the SiC-AC porous structure calculated by the D–R equation (Table 1).

As follows from the nitrogen adsorption data on the porous structure, SiC-AC is a mostly microporous material with a sufficiently high volume of micropores described by a narrow pore size distribution. The peak of the pore size distribution is observed at a size which is commensurate with a two-fold xenon molecular dimension (2 × 0.41 nm [48]). Therefore, according to the IUPAC classification [46], SiC-AC is a nanoporous material, which is efficient for gas storage and capture applications. In particular, one can expect that SiC-AC exhibits a high performance as an adsorbent for xenon. For example, a xenon-concentrating unit of the medicine xenon recycling system implies an efficient carbon adsorbent, with an average pore width between 0.6 and 1.2 nm, and a micropore volume exceeding 0.4 cm^3^/g [13]. However, there is no direct correlation between the adsorption performance of an adsorbent and its structural and energy characteristics [49]. Textural factors, including morphological features, pore size distribution, the density and nature of heteroatoms derived from a precursor, and activation procedure [50], should be taken into account. The latter factor is especially relevant for the adsorption of polarizable xenon.

**Figure 4:**
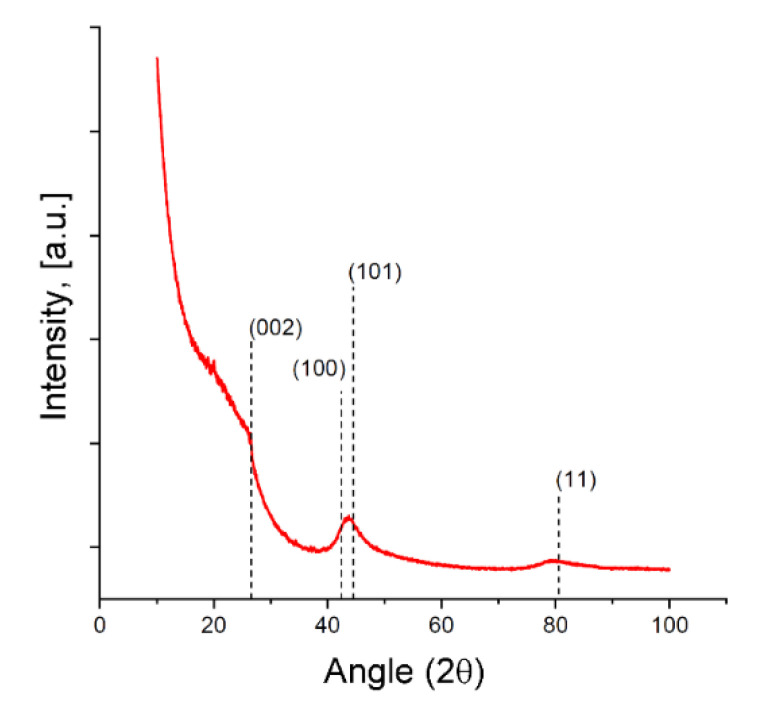
X-ray diffraction (XRD) patterns for the SiC-AC carbon adsorbent. The primary diffraction peaks of graphite are shown by dashed lines.

The XRD pattern for the SiC-AC adsorbent is shown in Figure 4, and the weakly exhibited and blurred diffraction peaks associated with the graphite (002) and (11) planes are indicative of a disordered carbon phase. The diffraction peak of the (100) graphite plane at 2θ ~ 42° can be attributed to a more ordered graphite-like phase.

It was found [51,52] that reliably estimating the dimensions and lattice spacing of graphitic crystallites smaller than 2–3 nm from the width and position of the 002 diffraction peak is not correct due to their strong dependence on the particular structure and morphology of the graphene layer stacks and surface termination. All these features of the XRD pattern point out that the SiC-AC carbon adsorbent lost the crystal structure of silicon carbide. Similar results were reported recently for the carbon adsorbents derived from silicon, titanium, and aluminum carbides subjected to treatment by electrical resistance heating in a chlorine gas atmosphere at temperatures increased from 1000 to 1500 °C [53]. A very intensive low-angle scattering gave evidence of a high density of pores [54], which is consistent with the data on the porous structure of the SiC-AC adsorbent (see Table 1).

The data listed in Table 2 indicate that the elemental composition of the SiC-AC adsorbent determined from the SEM-EDX data is not as diverse as that of activated carbons prepared from peat [55] or coconut shell [56].

The surface layer of the adsorbent consists mainly of carbon, and it also contains oxygen. The presence of insignificant amounts of Si and Cl heteroatoms in SiC-AC can be attributed to the precursor (silicon carbide) and thermochemical leaching in a chlorine flow. One can expect that heteroatoms, especially O, contribute to the inhomogeneous distribution of the energies of adsorption sites of the SiC-AC adsorbent.

**Figure 5:**
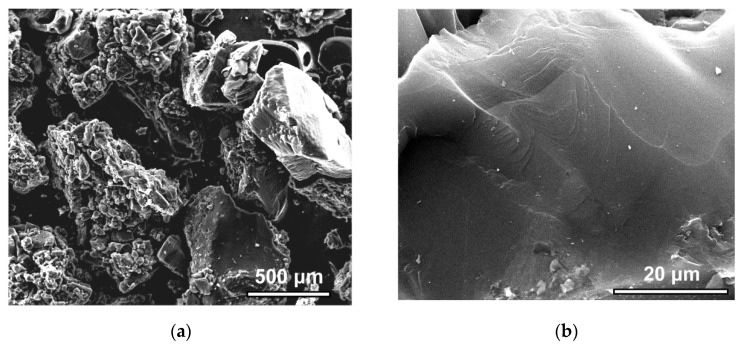
SEM images of the SiC-AC surface at a scale bar of 500 μm (**a**) and 20 μm (**b**).

The SEM images (Figure 5a,b) at different magnifications allowed for the qualitative characterization of the microstructure of the SiC-AC carbon adsorbent.

The SiC-AC adsorbent has a heterogeneous structure composed of dense particulates of different sizes and shapes (Figure 5a). Each particle has a smooth surface with sparse cavities and chips, while no macropores, which can serve as transport macropores, were found (Figure 5b).

### 3.2. Adsorption- and Temperature-Induced Deformation of the SiC-AC Adsorbent

**Figure 6:**
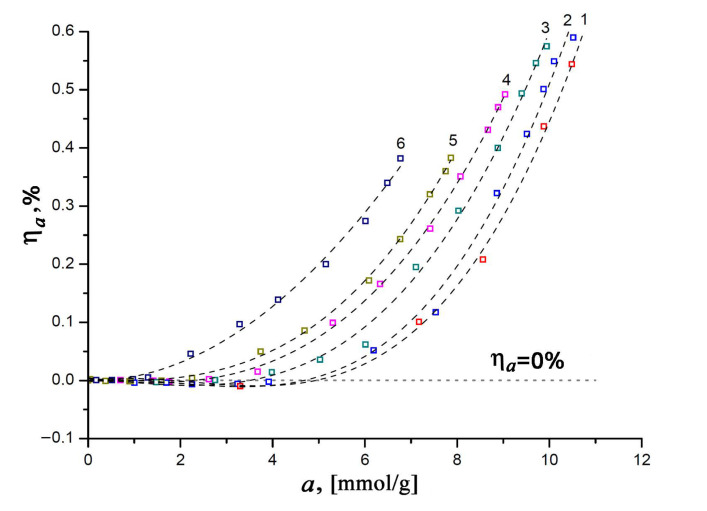
Relative linear deformation of the SiC-AC adsorbent versus Xe adsorption value at temperatures *T*, K: 1—216; 2—243; 3—273.15; 4—313; 5—353; and 6—393. Symbols are the experimental data; a dashed straight line indicates a zero variation in the size of the SiC-AC adsorbent; and dashed curves are the spline approximation.

As mentioned above, the correct thermodynamic characteristics of an adsorption system can only be determined by taking into account the effects associated with the non-ideality of a gaseous phase and the non-inertness of an adsorbent. With this in mind, let us consider the experimental dependencies of the relative linear deformation of the SiC-AC adsorbent on the value of xenon adsorption measured at various temperatures (see Figure 6).

It was found that the changes in the size of the SiC-AC adsorbent induced by adsorption appeared to be reversible upon desorption. As is seen from Figure 6, the value of relative deformation at the constant amount of adsorbed Xe changes with temperature. It is quite evident that within a temperature range of 216 to 353 K (curves 1–5, see Figure 6) in the course of adsorption, the SiC-AC adsorbent exhibits a non-monotonic deformation, starting from contraction at low adsorption values (for example, η*_a_* < 0 for *a* < 5 mmol/g, curve 1) and turning into expansion as the xenon pressure increases (η*_a_* < 0, for *a* > 5 mmol/g, curve 1). Such a non-monotonic character of the adsorption-induced deformation is inherent to microporous adsorbents, including activated carbons [57,58,59,60,61,62,63,64,65,66,67], zeolites [68], and MOF [69,70]. Neimark and Grenev proposed a theoretical approach [71] based on Polanyi’s potential adsorption theory [72], which was later elaborated by Dubinin and coworkers into the theory of volume filling of micropores [14,15]. Referring to [71], the opposite signs of the effects of the adsorption capacity of an adsorbent (or maximal pore volume filled at saturation pressure) and adsorption energy variations with deformation are responsible for the observed non-monotonic character of the adsorption-induced deformation in microporous adsorbents. At low pore loading, pores are contracted due to attractive interactions between xenon and SiC-AC (energy variations), and sparse xenon molecules serve as “couplings” between opposite pore walls. At 216 K, when the value of xenon adsorption is 3.3 mmol/g, curve 1 in Figure 6 reaches a minimum, which corresponds to the maximal contraction of the SiC-AC sample: η*_a_* = −0.009%. However, then the contribution from volume variation dominates at sufficiently high pressures, leading to pore expansion: the pores become populated with xenon molecules, which repel each other, subjecting the pore walls to positive stress [71]. The maximum relative expansion of SiC-AC induced by xenon adsorption reaches 0.59% (curve 2, Figure 6). It should be noted that SiC-AC exhibited somewhat lower negative and positive deformation compared to ACs prepared from other raw materials. For example, the carbon adsorbent prepared from furfural-based polymer exhibited the highest compression of 0.49% and expansion of 0.6% upon benzene adsorption at 293 K [60]. This can be attributed to the high stiffness of the SiC-AC adsorbent inherited from its precursor [73].

**Figure 7:**
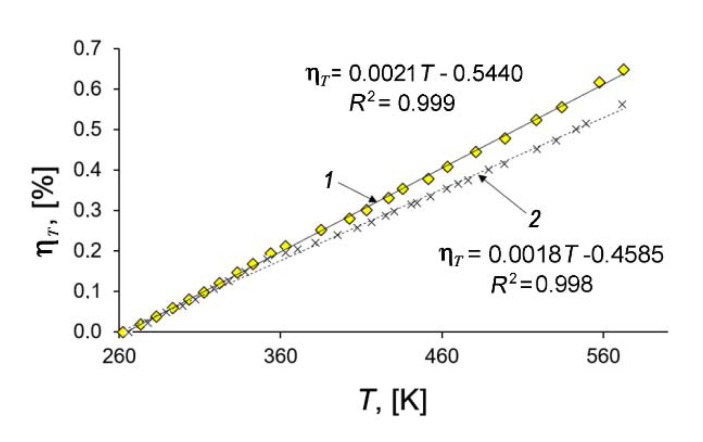
Relative length changes of the SiC-AC adsorbent (1) and quartz mockup (2) with temperature. Symbols are experimental data; solid straight lines are the linear approximations.

Figure 7 shows that the temperature dependencies of deformation of the SiC-AC adsorbent (1) and quartz mockup (2) are well approximated by linear functions within the 260 to 575 K range. By differentiating the linear approximation function according to Equation (4) and taking into account both the calibration data for the quartz mockup (line 2, Figure 7) and the thermal correction of the dilatometer [41], we evaluated the thermal expansion factor of the studied adsorbent from Equation (4): α = (3 ± 0.15)×10^−6^ K^−1^. It should be mentioned that the thermal expansion of SiC (6H, hexagonal) measured by XRD along the principle axes at ambient pressures was reported between 3.2 × 10^−6^ K^−1^ at 300 K and 6.0 × 10^−6^ K^−1^ at 1750 K [74].

### 3.3. Xenon Adsorption on the SiC-AC Adsorbent

**Figure 8:**
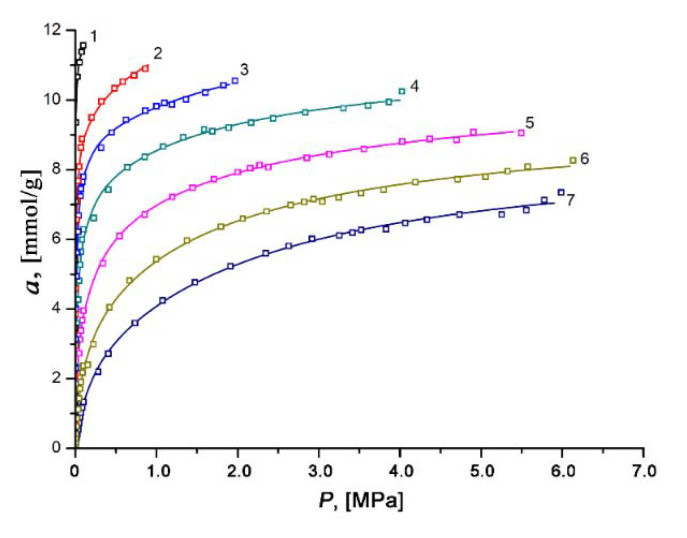
Dependencies of absolute xenon adsorption on the SiC-AC adsorbent on pressure at temperatures, K: 1—178; 2—216; 3—243; 4—273.15; 5—313; 6—353; and 7—393. Symbols are experimental data; solid lines are the approximation results by Equation (6) proposed in [75].

The experimental isotherms of xenon adsorption on the SiC-AC adsorbent measured over the temperature range of 178 to 393 K and at pressures up to 6.5 MPa remained in Type I (see Figure 8). It was found that xenon adsorption was reversible and increased with pressure. The isotherms of xenon adsorption were approximated using a formula derived by Bakaev using the statistical thermodynamics for a model of adsorption in cavities as quasi-independent subsystems of the grand canonical ensemble [75]:

(6) $$a\left(P\right)=\frac{k_{0\;}\left(k_{1}P+2k_{2}P^{2}+3k_{3}P^{3}\right)}{1+k_{1}P+k_{2}P^{2}+k_{3}P^{3}}$$

where *k*_0_ characterizes an adsorption system, *k*_1_, *k*_2_, *k*_3_ are the temperature-dependent and numerically adjusted coefficients, and *P* is the equilibrium pressure expressed in Pa. This formula fitted the experimental adsorption data for various gases in microporous adsorbents including zeolites [75], polymer sorbents [76], and activated carbons of different origins [55,56,77].

In our case, the regression error was not more than 3%.

For Xe storage, the SiC-AC sorption properties are competitive with those of the porous materials (MOF, polymer cage, zeolite, and AC) described in the literature and summarized in Table 3.

Two factors determine the efficiency of porous materials for Xe adsorption: (1) localized binding sites (open-metal sites, more polarizing organic groups); and (2) suitable pore size. Keeping in mind the textural properties of SiC-AC (see Table 1 and Table 2), one can conclude that its adsorption capacity for Xe is primarily determined by a sufficiently high volume of homogeneous slit-like micropores with a width commensurate the size of a xenon molecule. Some contribution to the Xe adsorption value may also be expected from oxygen heteroatoms due to the Xe–O interactions caused by the large polarizability [81,83].

**Figure 9:**
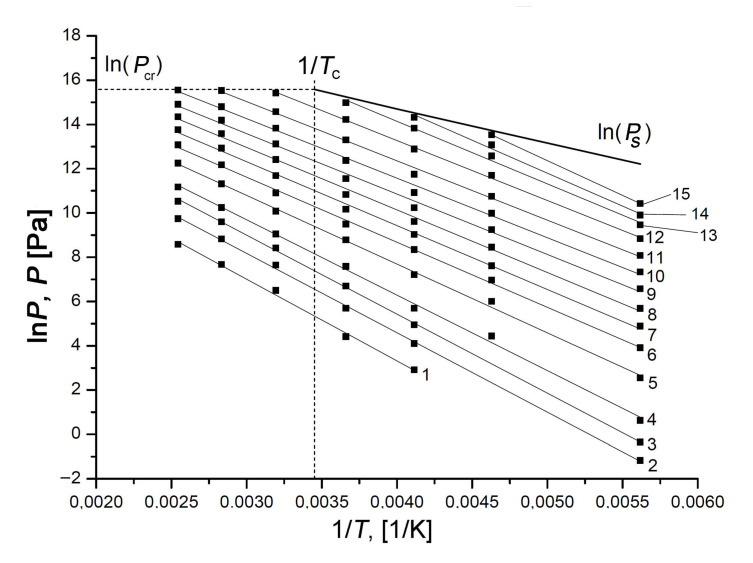
The isosteres of Xe adsorption in SiC-AC at the Xe adsorption values, *a*, mmol/g: 0.1 (1); 0.3 (2); 0.6 (3); 1.0 (4); 2.0 (5); 3.0 (6); 4.0 (7); 5.0 (8); 6.0 (9); 7.0 (10); 8.0 (11); 9.0 (12); 9.8 (13); 10.3 (14); and 10.8 (15). The experimental data (symbols) are approximated by the linear functions (solid straight lines). The bold line shows ln*P*_s_, where *P*_s_ is the saturated vapor pressure; the dashed lines correspond to the critical pressure and temperature of xenon.

We used a set of experimental adsorption isotherms measured within wide ranges of temperature and pressures to plot the isosteres in ln(*P*) = *f*(1/*T*) coordinates for different values of Xe adsorption on the SiC-AC adsorbent, which enabled us to explore the temperature dependence of Xe adsorption under sub- and supercritical conditions (see Figure 9).

The isosteres of Xe adsorption on the SiC-AC adsorbent shown in Figure 9 are well approximated by straight lines within the entire range of temperatures, including supercritical conditions, i.e., at *T* > *T*_cr_ = 289.73 K. A set of experimental data is available to date, revealing linear adsorption isosteres of vapors and gases [80,84,85,86,87,88,89,90,91,92,93,94,95], including nitrogen [91], methane [94], noble gases such as xenon [80,88], krypton [88,92,93], and argon [89], adsorbed in microporous activated carbons, zeolites. It was reported in [57,90,93,94,95,96] that the linearity of adsorption isosteres extended to the range of the compressed liquid state could be considered as intrinsic to adsorption in micropores and indicative of a particular state of an adsorbed substance. Thus, the observed linearity of the isosteres over the entire temperature range enabled us to suggest that xenon molecules in micropores of SiC-AC are in this highly dispersed state in a strong adsorption field generated by the opposite pore walls, which prevents them from the formation of a liquid phase in the porous space of the adsorbent. We used the experimental linear isosteres for calculating the thermodynamic state functions of the Xe/SiC-AC adsorption system over wide ranges of temperatures and pressures.

### 3.4. Thermodynamic Behaviors of the Xe/ SiC-AC Adsorption System

#### 3.4.1. Differential Molar Heat of Absolute Xe Adsorption on the SiC-AC Carbon Adsorbent

The analysis of the heat of adsorption enabled one to judge the strength of intermolecular interactions between adsorbate molecules and a porous solid. There are two established methods for evaluating heats of adsorption. The first method is by direct measurements using adsorption calorimetry, which requires a purpose-built setup [97,98]. The second method is indirect since it employs the isosteres of adsorption. The equation was derived with the usual approximation of an ideal gas, and it assumes that the adsorbed phase volume is negligible compared to a gaseous phase [35].

Following the definition given by Hill in [35], the differential isosteric molar heat of adsorption (*q*_st_) can be calculated as the difference between the molar enthalpy of the gas phase, *h*_g_, and the differential enthalpy of the adsorption system, *h*_1_:

(7) $$q_{\mathrm{st}}\equiv h_{g}-{\left(\frac{\partial H_{1}}{\partial N_{a}}\right)}_{T}=h_{g}-h_{1},$$

where *N_a_* is the amount of adsorbed substance determined as a total content.

When employing the variable replacement method in thermodynamics of adsorption equilibrium, Bakaev offered an equation for calculating the differential molar isosteric heat of adsorption, which involves the terms related to the non-ideality of a gaseous phase and the non-inertness of an adsorbent [32,57,99]:

(8) $$q_{\mathrm{st}}\;=\;-R\cdot Z\cdot {\left[\frac{\partial \left(lnP\right)}{\partial \left(1/T\right)}\right]}_{a}\cdot \left[1-{\left(\frac{\partial V_{a}}{\partial a}\right)}_{T}/\nu_{g}\right]-{\left(\frac{\partial P}{\partial a}\right)}_{T}\cdot \left[V_{a}-T\cdot {\left(\frac{\partial V_{a}}{\partial T}\right)}_{a}\right]$$

where *R* is the universal gas constant [J/(mol·K]; *Z* = *P·*ν_g_/(*RT*) is the coefficient of compressibility of an equilibrium gas phase at pressure *P* [Pa] and temperature *T* [K]; ν_g_ is the specific gas phase volume [m^3^/kg]; *V_a_* = *V*_0_(*P,T*)/*m*_0_ is the reduced volume of the adsorbent/adsorbate system [cm^3^/g]; and *V*_0_ and *m*_0_ are the volume and mass of the regenerated adsorbent, respectively. Thus, Bakaev’s equation (7) fully takes into account isothermal adsorption-induced deformation (∂*V_a_*/∂*a*)*_T_*, temperature isosteric deformation (∂*V_a_*/∂*T*)*_a_*, the slopes of the isotherm of adsorption (∂*P*/∂*a*)*_T_* and isosteres [∂ln*P*/∂(1/*T*)]*_a_*, and the non-ideality of a gas phase *Z*.

Another definition of the differential isosteric molar heat of absolute adsorption also follows from the rigorous application of the first law of thermodynamics to an adsorption system, which is compliant with the volumetric method [99,100,101,102] and is calculated as the difference between the molar enthalpy of the gas phase, *h_g_*, and the differential internal energy of adsorption system, (∂*E*_1_/∂*N_a_*)*_T_*:

(9) $$q_{d}\equiv h_{g}-{\left(\frac{\partial E_{1}}{\partial N_{a}}\right)}_{T},$$

When analyzing these functions, it is important to bear in mind that the molar enthalpy depends on temperature *T* and pressure *P* of the gaseous phase, while (∂*E*_1_/∂*a*)*_T_* can be regarded as an indirect estimate of the adsorbent–adsorbate interactions.

The differential internal energy of an adsorption system can also be derived using the replacement of variables considering the non-ideality of a gaseous phase and the non-inertness of an adsorbent [103]. Therefore, the differential heat of adsorption is expressed as follows [103]:

(10) $$q_{d}=\;-R\cdot Z\cdot {\left[\frac{\partial \left(lnP\right)}{\partial \left(1/T\right)}\right]}_{a}\cdot \left[1-{\left(\frac{\partial V_{a}}{\partial a}\right)}_{T}/\nu_{g}\right]+T{\left(\frac{\partial P}{\partial a}\right)}_{T}\cdot {\left(\frac{\partial V_{a}}{\partial T}\right)}_{a}+P{\left(\frac{\partial V_{a}}{\partial a}\right)}_{T}.$$

It is obvious that the difference between these two heats is caused by the non-ideal conditions and depends on the slope of the isotherm of adsorption (∂*P*/∂*a*)*_T_* and adsorption-induced isothermal deformation (∂*V_a_*/∂*a*)*_T_*.

Attempts have been made to compare the isosteric heat measurements using the calorimetric methods with the theoretical predictions based on different models [104,105,106]. An average difference of approximately 2 kJ/mol was found between the values evaluated by Claysius–Clapeyron type equations and the results of calorimetry experiments, even for low-pressure adsorption processes [104]. It was revealed that the differential heat of adsorption, which was measured experimentally (calorimetrically), approaches *q*_d_ when the amount of gas injected into a calorimeter tends to zero (∆*N* *→* 0) [101]. Some experimental data provided evidence for this statement [97,102,106]. Recently, Tian et al. reported an expression for the heat of adsorption derived from Equation (9) using gas fugacity and validated the calculated values of the differential heat of adsorption with the data of grand canonical Monte Carlo simulations (GCMC) [102].

It should be noted that without consideration of the non-ideality of a gas phase (Z=1; $\left(\frac{\partial P}{\partial a}\right)\to \;0$) and the non-inertness of an adsorption system (η*_a_*=0, and η*_T_*=0) Equations (8) and (10) come to a well-known formula:

(11) $$q_{\mathrm{st}}^{0}\;=q_{d}^{0}=\;-R\cdot {\left[\frac{\partial \left(lnP\right)}{\partial \left(1/T\right)}\right]}_{a}.$$

**Figure 10:**
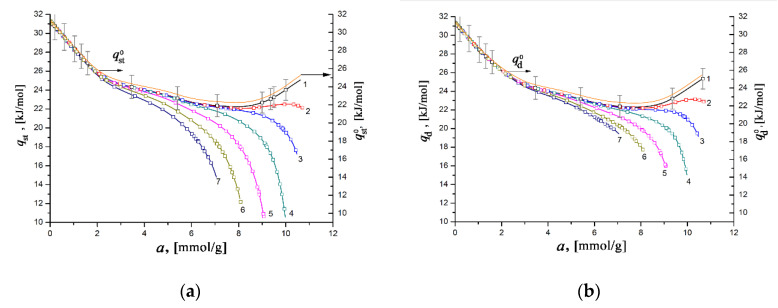
The differential isosteric molar heat of xenon adsorption *q*_st_ (**a**) and differential molar heat of adsorption *q*_d_ (**b**) versus the value of absolute Xe adsorption in the SiC-AC adsorbent at temperatures, K: 178 (1), 216 (2), 243 (3), 273 (4), 313 (5), 353 (6), and 393 (7). Symbols indicate the data calculated from the Xe adsorption data using Equations (8) and (10), respectively. Solid lines are the results of curve fitting. The yellow curves (right axis) correspond to $q_{\mathrm{st}}^{0}$ and $q_{d}^{0}$ calculated by Equation (11) suggesting Z = 1, $\left(\frac{\partial P}{\partial a}\right)\to \;0$; η*_a_*= 0%, and η*_T_*= 0%. The error bar is ±5%.

Figure 10 illustrates the dependencies of the differential heats of xenon adsorption in the SiC-AC adsorbent, *q*_st_ = *f*(*a*) (a) and *q*_d_ = *f*(*a*) (b), within a wide range of temperatures, calculated from Equations (8) and (10) using the isotherms, isosteres of xenon adsorption (Figure 9), data on the adsorption- and temperature-induced deformations (Figure 6 and Figure 7), and the handbook data on xenon compressibility, which varied from 0.59 to 1.0 [107].

As seen from Figure 10a,b, the difference between the differential heats of adsorption derived from enthalpy and internal energy becomes noticeable for *a* > 4–5 mmol/g. However, the behaviors of *q*_st_ = *f*(*a*) and *q*_d_ = *f*(*a*) are almost identical to each other and are typical for gas adsorption in microporous adsorbents containing high-energy adsorption sites. For example, the resembling dependencies *q*_st_ versus *a* were reported previously for xenon adsorbed in the MOF material HKUST-1 [27], neon in titanium dioxide [108] and HKUST-1 [27], and krypton in AC [92,93] and HKUST-1 [27]. At a pressure as low as 100 Pa, the high values of *q*_st_ and *q*_d_ of ~ 30–31 kJ/mol are observed when gas molecules fill a significant part of the micropores by binding to high-energy adsorption sites. It is evident that at this stage, the value of the differential heat of adsorption is determined by the density of these high-energy sites, which are surface heteroatoms (in our case, primarily O), or micropores with dimensions commensurate to that of Xe molecules.

With an increase in the Xe pressure and, consequently, pore loading, the high-energy adsorption sites are entirely occupied, and the heat of adsorption is determined by the contribution from the adsorbate–adsorbate interactions. Thus, the variations of both quantities *q*_st_ and *q*_d_ with the value of Xe adsorption (Figure 10a,b) are caused by the transformation in the mechanism of the Xe adsorption process, from binding with the high-energy adsorption sites to the volume filling of micropores accompanied by the increased contribution from adsorbate–adsorbate interactions.

As follows from Figure 10a,b, at the early stage of Xe adsorption in the SiC-AC adsorbent (*a* ≤ 3–4 mmol/g), both isosteric heats of adsorption do not depend on temperature. When the pore loading increases, the curves of both functions *q*_st_ = *f*(*a*) and *q*_d_ = *f*(*a*) diverge and form a “fan”. The observed divergence is caused by the difference in the contributions from the non-inertness of the SiC-AC adsorbent (see Figure 6 and Figure 7), slopes of isosteres of adsorption, and coefficient of compressibility of the gaseous phase. The contributions from the non-ideality of the gaseous phase and non-inertness of the SiC-AC adsorbent manifest as a deviation of the curves *q*_st_ = *f*(*a*) and *q*_d_ = *f*(*a*) from that for Z = 1 and η*_a_*= 0% and η*_T_*= 0% starting from *a* = 2 and 1.5 mmol/g, respectively.

#### 3.4.2. Differential Molar Entropy of Xe Adsorption on the SiC-AC Adsorbent

**Figure 11:**
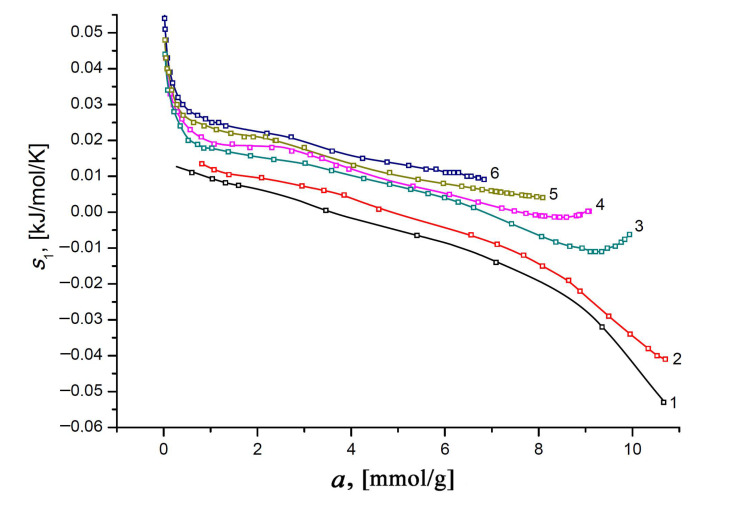
The differential molar entropy of the Xe/SiC-AC adsorption systems versus the values of absolute Xe adsorption at temperatures, K: 178 (1), 216 (2), 273 (3), 313 (4), 353 (5), and 393 (6). Symbols indicate the results of calculations by Equations (10) and (12); solid lines are the smoothing spline curves.

The differential entropy of an adsorption system, *s*_1_, is an essential thermodynamic state function associated with the degrees of motion of molecules in porous space: translational, rotational, and vibrational modes. We calculated the value of *s*_1_ for the Xe/SiC-AC adsorption system using a general formula [101]:

(12) $$s_{1}\;=\;s_{g}-\frac{q_{d}}{T}$$

and plotted the results of the calculations for different temperatures as a function of the Xe adsorption value (Figure 11).

Obviously, the transition of mobile Xe molecules from a disordered gaseous state to a bound state at the high-energy adsorption sites in micropores is accompanied by a decrease in their molecular mobility. Therefore, the differential molar entropy can be informative in studying porous materials with a large number of various adsorption sites [109]. With this in mind, we can ascribe a significant decrease in the differential entropy observed at the early stage of xenon adsorption (*a* < 0.5 mmol/g) to the localized adsorption of xenon molecules on the various adsorption sites. In the range of *a* from 1 to 7 mmol/g, the rate of fall of the curve *s*_1_(*a*) decreases, reflecting the “saturation” of the different adsorption sites. The further trend of the curve on the adsorption value is determined by an increase in the contribution of the Xe–Xe interactions, leading to the formation of Xe molecular associates and an increase in the intensity of random temperature-dependent molecular motions (compare curves 1 and 3–7). The rise portions of the curves *s*_1_(*a*) reflect an increase in the intensity of the molecular mobility of xenon molecules in the molecular associates (or clusters) at elevated temperatures.

#### 3.4.3. Differential Molar Enthalpy of Xe Adsorption on the SiC-AC Adsorbent

**Figure 12:**
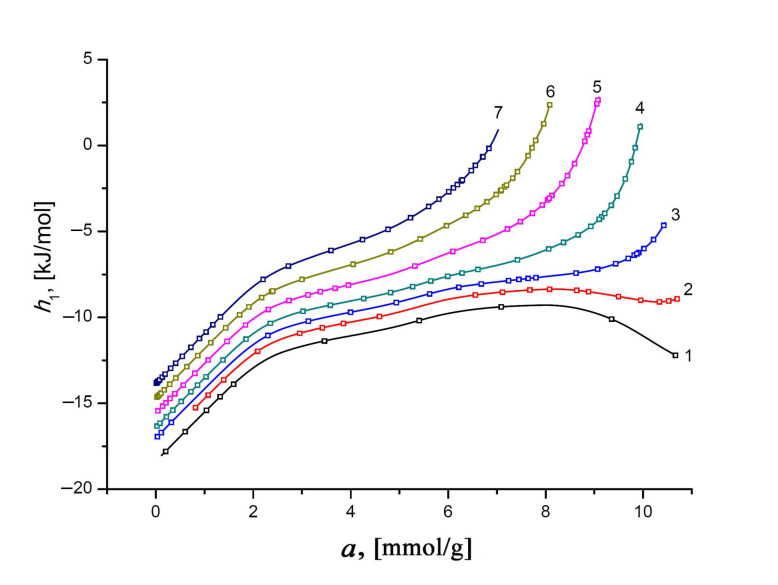
The differential molar enthalpy of the Xe/SiC-AC adsorption system versus the value of absolute Xe adsorption, *a,* at temperatures, K: 178 (1), 216 (2), 243 (3), 273 (4), 313 (5), 353 (6), and 393 (7). Symbols indicate the data calculated from the Xe adsorption data using Equations (8) and (13); solid lines are the smoothing spline curves.

Figure 12 represents the differential molar enthalpy of the Xe/SiC-AC adsorption system, *h*_1_, calculated according to the general formula [35]:

(13) $$h_{1}\;=\;h_{g}-q_{\mathrm{st}},$$

The negative values of enthalpy *h*_1_ of Xe in the SiC-AC adsorbent are conditioned by choosing a reference level accepted for the standard state of the gas phase—*h*_g_. Similar to the isosteric heat of adsorption, the plots of *h*_1_(*a*) display two features, caused by the adsorbent properties, which affect the state of adsorbed Xe molecules. First, an increase in *h*_1_ at low pore loading is a consequence of a heterogeneous distribution of adsorption sites (heteroatoms and narrow pores). Second, an increasing contribution from the Xe–Xe interactions compared to the Xe–SiC-AC bindings is responsible for the changes in the rate of the *h*_1_(*a*) curves.

**Figure 13:**
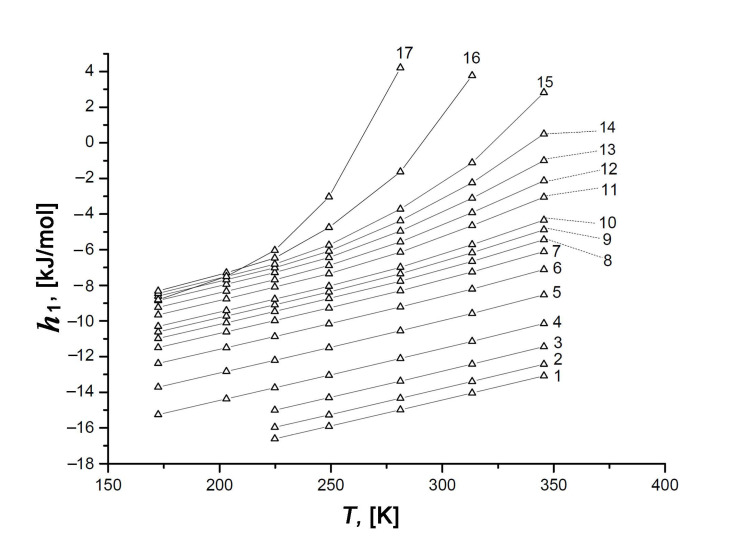
Temperature dependence of the differential molar enthalpy, *h*_1_, of the Xe/SiC-AC adsorption systems at the values of adsorption *a*, mmol/g: 1—0.1 (1), 0.3 (2), 0.6 (3), 1.0 (4), 1.5 (5), 2.0 (6), 2.5 (7), 3.0 (8), 3.5 (9), 4.0 (10), 5.0 (11), 5.5 (12), 6.0 (13), 6.5 (14), 7.0 (15), 8.0 (16), and 9.0 (17). Symbols are the results of calculations by Equation (13), lines are the spline approximations.

As exemplified by the plots *h*_1_ = *f*(*T*) in Figure 13 for the different values of Xe adsorption, the variation in the ratio of the impacts from Xe–Xe and Xe–SiC-AC interactions upon the adsorption process depends on the temperature. Indeed, the temperature invariance *q*_st_ ≠ *f*(*T*), which is observed for the Xe/SiC-AC adsorption system, and the linear character of the function *h*_g_(*T*) determine the almost linearly increasing curve *h*_1_(*T*) at low pore loading. However, the linear behavior of *h*_1_(*T*) changes with the increase in the Xe adsorption value (see curves 10–17, Figure 13), which is attributed to the temperature-dependent compressibility of the gas phase Z and production (∂*P*/∂*a*)⋅*V_a_* in Equation (7) for *q*_st_ (see Figure 10).

#### 3.4.4. Differential Molar Isosteric Heat Capacity of the Xe/SiC-AC Adsorption System

The complete thermodynamic analysis of a Xe/Si-AC adsorption system involves the calculation of the specific heat capacity, *C_a_*, which depends not only on pressure and temperature but on the value of adsorption. In our calculations, we employed the Kirchhoff equation derived by the differentiation of Equation (13) over the temperature:

(14) $$C_{a}\;=\;{\left(\frac{\partial h_{1}}{\partial T}\right)}_{a}\;=\;{\left(\frac{\partial h_{g}}{\partial T}\right)}_{a}-\;{\left(\frac{\partial q_{\mathrm{st}}}{\partial T}\right)}_{a}.$$

As indicated above, at low values of pressure, the enthalpy of an adsorption system, *h*_1_, increases with the temperature almost linearly since *q*_st_ *≠ f*(*T*). The enthalpy of a gaseous phase, which is expressed as follows:

(15) $$h_{g}\;=\;h_{0}+\int^{\;}C_{P}dT,\;$$

is a linear function of temperature, and *h*_0_ is a reference level of the enthalpy.

**Figure 14:**
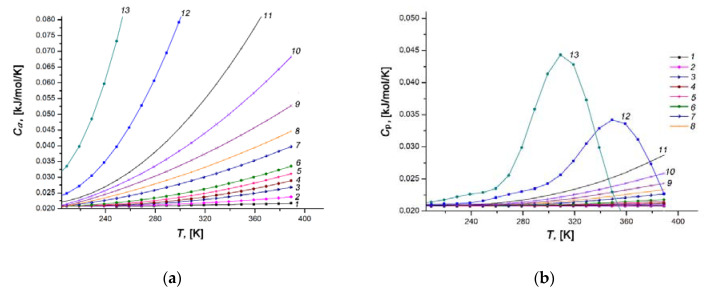
Temperature dependences of the differential molar isosteric heat capacities of the Xe/SiC-AC adsorption system (**a**) and the xenon gaseous phase (**b**) at the values of xenon adsorption, a, mmol/g: 0.1 (1), 1.5 (2), 2.5 (3), 3.0 (4), 3.5 (5), 4.0 (6), 5.0 (7), 5.5 (8), 6.0 (9), 6.5 (10), 7.0 (11), 8.0 (12), 9.0 (13).

Figure 14a,b shows the temperature dependencies of the differential molar isosteric heat capacities of the Xe/SiC-AC adsorption system at a constant adsorption value *a*, *C_a_*(*T*), and the heat capacity of the Xe gaseous phase at constant pressure *C_p_(T)*, respectively.

At low pressures and temperatures, it is valid that:

(16) $${\left(\frac{\partial q_{\mathrm{st}}}{\partial T}\right)}_{a}\;\approx 0;\;{\left(\frac{\partial h_{g}}{\partial T}\right)}_{a}=C_{g}\approx C_{P},$$

and, hence, the differential isosteric molar heat capacity of the adsorption system approximately equals to the isobaric heat capacity of gaseous phase *C*_P_, which is consistent with the corresponding curves in Figure 14a,b.

As the pore loading increases, the rate of rise of *C*_a_(*T*) grows markedly (compare curves 1–6 with 7–13, Figure 14a). An analysis of a rich set of experimental data for gas (vapor)/microporous solid adsorption systems [57,80,110,111] revealed the existence of a local maximum of *C*_a_(*T*) at high temperatures and pressures. Therefore, one can expect a similar maximum of *C*_a_(*T*) for the Xe/SiC-AC adsorption system at some temperatures and pressures, which we only came near. Nevertheless, a tendency in the behaviors of *C*_a_(*T*) for the Xe/SiC-AC is consistent with that reported in [57,80,110,111]: the maximum (a growing section, in our case) became pronounced and shifted towards low temperatures with an increase in the Xe adsorption value. The achievement of the maximum heat capacity of a gas (vapor)/microporous solid adsorption system is attributed to the changes in the state of adsorbed molecules: from the bound state in a deep well of potential attractive energy (localized adsorption on the high energy adsorption sites) to the molecular associates (or clusters) characterized by relatively high kinetic energy. This conclusion agrees with the results of the numerical experiment for methane [95,112] adsorbed in microporous activated carbons.

## 4. Conclusions

The carbon adsorbent prepared from silicon carbide, SiC-AC, was examined to view the potential application for adsorption-based storage of xenon. The nitrogen adsorption measurements at 77 K revealed a developed microporosity of SiC-AC, which is described by a relatively homogeneous pore size distribution with a maximum at the pore size, which is commensurate with a Xe molecular dimension. The XRD and SEM data revealed that the SiC-AC adsorbent lost the crystallinity of silicon carbide and retained the trace amounts of Si, O, and Cl inherited from the precursor—silicon and activating agent—chlorine.

The analysis of Xe adsorption isotherms within the wide ranges of temperatures and pressures revealed a relatively high Xe adsorption capacity of the SiC-AC microporous material, which is comparable with the other efficient adsorbents. The efficiency of the SiC-AC was primarily ascribed to a sufficiently high volume of slit-like micropores with a size of no less than one Xe molecule, and, to a lesser degree, to the presence of some amount of oxygen.

The dilatometric measurements within the wide ranges of temperatures and pressures disclosed a contraction of the SiC-AC adsorbent at the early stages of Xe adsorption followed by its expansion upon further xenon adsorption. This variation in the sign of deformation is attributed to a variation in the relative contribution of Xe–Si-AC and Xe–Xe intermolecular interactions with increasing pore loading. The adsorption-induced deformation, the data on the thermal expansion of SiC-AC, and the non-inertness of the Xe gaseous phase were considered in calculating the thermodynamic parameters of the Xe/Si-AC adsorption system from the experimental isosteres of Xe adsorption at pressures up to 6 MPa and temperatures within the range of 178 to 393 K using the general thermodynamic equation by Bakaev. It was found that the dependencies of thermodynamic state functions, namely, differential molar isosteric entropy, enthalpy, and heat capacity for the Xe/SiC-AC adsorption system, on temperature and the value of Xe adsorption reflect the changes in the state of adsorbed Xe molecules upon the adsorption process in the microporous SiC-AC carbon: from the localized state near the high-energy adsorption sites to the adsorption molecular associates (or clusters), which rearranged close to saturation.

Finally, these findings on the thermodynamic behaviors of the Xe/microporous carbon material adsorption system can yield useful predictions about heat effects, which accompany the Xe concentrating process via physical adsorption in real recycling and Xe/Kr separation systems.

## Figures and Tables

**Table 1:** The porous structure parameters of the SiC-AC adsorbent calculated from the isotherm of nitrogen vapor adsorption/desorption at 77 K by the D-R, Brunauer–Emmet–Teller (BET), and Kelvin [47] equations.

*W*_0_, cm^3^/g	*E*_0_(N_2_), kJ/mol	*E*_0_(C_6_H_6_) ^1^, kJ/mol	*x*_0_, nm	*S*_BET_, m^2^/g	*W*_total_, cm^3^/g	*W*_meso_, cm^3^/g
0.48	9.6	29.2	0.41	1110	0.50	0.02

^1^*E*_0_(C_6_H_6_) is the standard characteristic energy of adsorption calculated from the adsorption isotherm of standard benzene vapor at 293 K, *E*_0_(C_6_H_6_) = *E*_0_(N_2_)/β; β = 0.33 is the coefficient of similarity; *x*_0_ = 12/*E*_0_(C_6_H_6_) [14,15].

**Table 2:** The atomic and weight percent of the elemental composition of the SiC-AC adsorbent.

Units	Elements			
	C	O	Si	Cl
wt. %	93.89	4.20	1.04	0.86
at. %	96.02	3.23	0.46	0.30

**Table 3:** Comparison of the low-pressure Xe adsorption values in MOF (No. 1–3), porous organic cage (No. 4), zeolites (No. 5–7), and activated carbons (No. 8–10).

No	Adsorbent	Pore Size, *D*, nm	Xe Adsorption, mmol/g; (*T*,*P*-Conditions)	Ref.
1	Co^+2^-CPM-6	0.54 ≤ *D* ≤ 0.86	3.2 (298K, 1 bar) data	[78]
2	PCN-12	0.78; 0.77; 1.45	5.4 (298 K, 1 bar) data	[79]
3	NiDOBDC	1.1	4.3 (298 K, 1 bar)	[79]
4	CC3	0.36	2.2 (298, 1 bar)	[25]
5	Zeolite Koestrolith 13X-K2	0.9	4.8 (303 K, 0.9 bar)	[17]
6	Zeolite NaX	0.62	2.7 (295 K, 1 bar)	[80]
7	Ag-doped ZSM-5	0.5	1.8 (298 K, 1 bar)	[81]
8	SorboNorit B3	0.8	3.17 (303 K, 0.9 bar)	[17]
9	Carbon-Zx	0.2 ≤ *D* ≤ 2	4.42 (298 K, 1 bar)	[82]
10	SiC-AC	0.82	3.9 (313 K, 1 bar)	This work

## Data Availability

Not applicable.
